# Vehicle Counting in Video Sequences: An Incremental Subspace Learning Approach

**DOI:** 10.3390/s19132848

**Published:** 2019-06-27

**Authors:** Leonel Rosas-Arias, Jose Portillo-Portillo, Aldo Hernandez-Suarez, Jesus Olivares-Mercado, Gabriel Sanchez-Perez, Karina Toscano-Medina, Hector Perez-Meana, Ana Lucila Sandoval Orozco, Luis Javier García Villalba

**Affiliations:** 1 Instituto Politecnico Nacional, ESIME Culhuacan, Mexico City 04440, Mexico; 2 Group of Analysis, Security and Systems (GASS), Department of Software Engineering and Artificial Intelligence (DISIA), Faculty of Computer Science and Engineering, Office 431, Universidad Complutense de Madrid (UCM), Calle Profesor José García Santesmases, 9, Ciudad Universitaria, 28040 Madrid, Spain

**Keywords:** video processing, motion detection, incremental learning, Incremental PCA, traffic flow

## Abstract

The counting of vehicles plays an important role in measuring the behavior patterns of traffic flow in cities, as streets and avenues can get crowded easily. To address this problem, some Intelligent Transport Systems (ITSs) have been implemented in order to count vehicles with already established video surveillance infrastructure. With this in mind, in this paper, we present an on-line learning methodology for counting vehicles in video sequences based on Incremental Principal Component Analysis (Incremental PCA). This incremental learning method allows us to identify the maximum variability (i.e., motion detection) between a previous block of frames and the actual one by using only the first projected eigenvector. Once the projected image is obtained, we apply dynamic thresholding to perform image binarization. Then, a series of post-processing steps are applied to enhance the binary image containing the objects in motion. Finally, we count the number of vehicles by implementing a virtual detection line in each of the road lanes. These lines determine the instants where the vehicles pass completely through them. Results show that our proposed methodology is able to count vehicles with 96.6% accuracy at 26 frames per second on average—dealing with both camera jitter and sudden illumination changes caused by the environment and the camera auto exposure.

## 1. Introduction

Video surveillance systems are multistage computer vision systems capable of performing high end tasks [1]. Due to the increasing capabilities of hardware and software, the algorithms used to perform motion detection are getting better performance. However, there is still an increasing interest for developing new algorithms that are able to overtake limitations produced by human errors, since most of the systems cannot be checked automatically [2].

Video surveillance systems are broadly used in roads, banks, shops, schools, and other public places in order to protect social security [2,3]. At the present time, the challenge for these video systems is to provide accuracy and confidence for detecting motion in any scenario. Thus, many applications, for example traffic monitoring, are based on the unsupervised analysis of video sequences and are mainly focused in detecting and monitoring objects or elements of interest that appear in the scene [4]. In general, regions of interest in the scene are: Pedestrians, vehicles, and other elements that normally do not belong to the scene [5]. Motion detection is also a first step prior to performing more sophisticated tasks such as tracking or categorization of vehicles by their type.

Currently, the main methods for motion detection are optical flow, frame difference, and background subtraction [6]. Although all these pixel-based models support gradual and long-term illumination changes, they underperform in the presence of sudden ones [7]. Frame difference and background subtraction using Gaussian Mixtures methods often suffer from the phenomena called the foreground aperture problem, when parts of large moving homogeneous regions become part of the background instead of being selected as moving pixels—also, they tend to present ghost regions, which are the traces of the moving objects in some consecutive frames [8,9,10]. However, they are more robust to sudden illumination changes.

In this paper, we propose a different and novel methodology for detecting motion using incremental subspace learning in order to count vehicles passing over the roadway in video sequences. Our motivation lies in the fact that several applications require having knowledge about the vehicular flow. In the same way, a set of parameters needs to be known beforehand in order to implement Intelligent Transport Systems (ITSs) and to analyze traffic patterns [1,11]. Such parameters are: Levels of vehicular occupation, the vehicular average speed, and the number of vehicles transiting through each lane. These parameters also help to implement active traffic management solutions and to automate route planning [12,13]. On top of that, vehicle counting is one of the key technologies of intelligent transportation system and has received considerable interest in industry and research communities [14].

In the literature, there have been several approaches to addressing the problem of vehicle detection and counting. Some of them make use of traditional techniques such as background subtraction [13,15,16], filtering [17,18], Optical Flow [19], or Hidden Markov Models [13]. New emerging techniques based on Deep Learning such as Convolutional Neural Networks (CNNs), Recurrent Neural Networks (RNNs), and Long short-term memory (LSTM) networks have also been proposed [14,20,21,22,23]. The problem of vehicle detection and counting has been addressed from different points of view, such as top view [12,21], top-front view [15,16] and even on-road view for autonomous cars [18,24].

In addition, as stated in [22], the successful detection and classification of vehicle classes is essential to extract important traffic flow information needed by regulators, such as vehicle counts, average estimated speed of the vehicles, driver behavior (e.g., preferred lane usage), and violation of traffic rules (e.g., trucks using the high speed lanes).

Some local governments around the world are developing their own ITSs to address the new challenges of traffic crowding [25]. Video sources can provide overall information about the vehicles and are easy and cheap to both obtain and maintain [25]. On top of that, the use of already established video surveillance infrastructure in emerging countries makes it possible to implement ITSs with a minor or any need of additional hardware. Therefore, the use of economic and non-invasive sensors like video cameras allows us to identify useful parameters for ITSs while maintaining low operating costs [26].

The remainder of this paper is organized as follows: First, Section 2 presents the general idea and theory about Incremental PCA. Then, Section 3 presents our proposed methodology for counting vehicles, divided into three subsections: Motion detection using Incremental PCA, where we describe how we use this novel approach for detecting motion in video sequences; post-processing, where we describe all the steps used to improve regions of motion in the image obtained from the previous stage; and vehicle counting, where we show and implement our algorithms for performing the actual task. Subsequently, Section 4 presents the results of our methodology in different scenarios and under different testing conditions. Next, in Section 5 we discuss the limitations of our work and establish a comparison between similar works. Finally, Section 6 concludes this paper giving conclusions and discussing future works+.

## 2. Incremental Principal Component Analysis

Subspace learning model methods have been proposed in order to model the background in video sequences, as described in [27,28,29,30,31,32]. More specifically, such processes compute the mean image from a set of *N* frames available, then subtract the mean from all frames, and finally compute their eigenvalues and eigenvectors. As authors proposed in [27], the first most significant eigenvectors are used for computing the difference between the actual frame and the background frame previously modeled. These methods are based on a technique known as Batch PCA or Off-line PCA, which are not suitable for working with video sequences. However, novel approaches of incremental subspace learning have proven their effectiveness, allowing eigenbasis to be updated as soon as new frames are available, making them suitable for prolonged applications in real-time [33]. Nevertheless, as reported in [34], the vast majority of these methods do not take into consideration the update of the mean image as new video frames continue arriving.

The main idea of Incremental subspace learning is derived from Singular Value Decomposition (SVD). SVD provides the eigenvectors and eigenvalues sorted in descending order, so that the first eigenvectors provide the maximum variability of the data under analysis. The main contribution of this work is the idea that the first projected eigenvector contains the change of every new incoming frame, with respect to previous frames—in other words, the present motion in the frame. In [35], Levy and Lindenbaum proposed the Sequential Karhunen–Loeve (SKL) algorithm for efficiently updating the eigenbasis. However, SKL does not take into account the mean update as new training data arrive. In order to overcome this limitation, Lim and Ross presented a new Incremental PCA (IPCA) algorithm in [34] that properly updates the eigenbasis as well as the mean.

Now, supposing that $A=\{I_{1},...,I_{n}\}$ is the block of previous frames, $B=\{I_{n+1},...,I_{n+m}\}$ is the block containing the new incoming frames, and C=[AB] is their concatenation, *n* and *m* can be defined as the number of frames of each block *A* and *B*, respectively. Then, as formulated in [34], Algorithm 1 is implemented. First, computing the eigenvectors *U* and the eigenvalues Σ from the SVD of $A-{\bar{I}}_{A}$, where ${\bar{I}}_{A}$ is the average of the block from previous frames. Step No. 2 of Algorithm 1 shows the matrix $\hat{B}=B-{\bar{I}}_{B}$ where *B* is the block with incoming frames, and ${\bar{I}}_{B}$ is the mean considering the previous and new frames. Step No. 3 of Algorithm 1 represents in $\tilde{B}$ the components of *B* orthogonal to *U*, we can also represent *A* in terms of its eigenvalues and eigenvectors as $A=U\Sigma V^{T}$ and we can then express the concatenation of *A* and *B* as follows [34]:

(1) $$\left[A\;\tilde{B}\right]=\left[\begin{array}{cc} \Sigma & U^{T}B\\ 0 & {\tilde{B}}^{T}B \end{array}\right]\left[\begin{array}{cc} V^{T} & 0\\ 0 & I \end{array}\right].$$

Finally, making a substitution of $\hat{B}$ and $\tilde{B}$ we can represent *R*, and integrate the forgetting factor parameter *f*, which determines the influence of past observation by values in range [01]—a value close to 0 indicates no influence of previous frames; whereas a value close to 1 preserves the influence of all previous frames. Details about the formulation of this algorithm can be found in [34,35].

**Algorithm 1** Incremental PCA with mean update
1:Compute the mean vectors ${\bar{I}}_{B}=\frac{1}{m}\sum_{i=n+1}^{n+m}I_{i}$, and ${\bar{I}}_{C}=\frac{n}{n+m}{\bar{I}}_{A}+\frac{m}{n+m}{\bar{I}}_{B}$.2:Form the matrix $\hat{B}=\left[\left(I_{n+1}-I_{B}\right)\;...\;\left(I_{n+m}-I_{B}\right)\;\sqrt{\frac{nm}{n+m}}\left(I_{B}-I_{A}\right)\right]$.3:Compute $\tilde{B}=\mathrm{orth}\left(\hat{B}-UU^{T}\hat{B}\right)$ and $R=\left[\begin{array}{cc} f\Sigma & U^{T}\hat{B}\\ 0 & \tilde{B}\left(\hat{B}-UU^{T}\hat{B}\right) \end{array}\right]$.4:Compute the SVD of R: $R=\tilde{U}\tilde{\Sigma }{\tilde{V}}^{T}$.5:Finally, $U^{\prime }=\left[U\;\tilde{B}\right]\tilde{U}$ and $\Sigma^{\prime }=\tilde{\Sigma }$.6:Repeat for each new block of frames. Now with $A=U^{\prime }$.


## 3. Proposed Methodology

**Figure 1:**
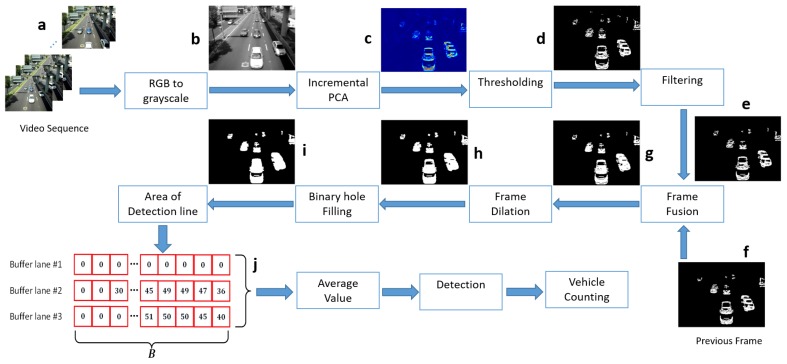
Proposed methodology block diagram, (**a**) video sequence, (**b**) gray scale image, (**c**) heat map of projected image, (**d**) binary image, as a result of thresholding process, (**e**) and (**f**) actual and previous frames binarized and filtered, (**g**) result of fusion from previous and actual frames, (**h**) dilated image, (**i**) result of filling hole process, (**j**) buffers representation for detection lines in different lanes.

In this section, we present the three main phases of our methodology for counting vehicles: The implementation of Incremental PCA for motion detection in video sequences; the post-processing steps needed for the image enhancement obtained from the first projected eigenvector, and finally our algorithm for performing the actual vehicle counting. These phases are summarized in Figure 1 and described in detail in the following subsections.

The block diagram of the proposed methodology consists of the input data, i.e., the **Video sequence**, the individual frames from the video sequence depicted in Figure 1a passes through the **RGB to grayscale** block in order to convert each frame from an RGB to a grayscale image Figure 1b Then, the grayscale frames are the input for the Incremental PCA block once the first eigenvector is used to project the actual grayscale image, the resulting image is shown in Figure 1c, this projected image uses the function heatmap to remark the variance in the pixels which represent motion, this projected image passes through the **Thresholding** block, in this block are thresholds based on statistic parameters (mean and standard deviation), it will set the pixels to “one” if these pixels belong to the foreground or movement; and set to “zero” if the pixels belong to the background—the results of the Thresholding block is the image in Figure 1d, but this image contain small groups of pixels which do not represent significant movement, and could be considered noise in the motion detection. To solve this issue, the **Filtering** block is implemented to eliminate those groups of pixels below a minimum size, relative to the size of the frame, the results are presented in Figure 1e. Moreover, this continuous process uses two main frames from the video sequence, the actual frame and the previous frame. Another important issue in motion detection is the foreground aperture problem, splitting large objects due to the similarity of the object’s internal pixels, so to overcome the object separation as a consequence of the miss detected motion in the internal pixel of the object, **Frame fusion** block was implemented, and OR operator wass applied in actual and previous frames (Figure 1f), the overlap images form a single object, avoiding the typical split in large motion objects, the result is shown in Figure 1g. Now some lines in the object are very thin, in order to maintain those lines, the Frame Dilation block is implemented, the results are shown in Figure 1h. In order to avoid double counting of the same object, the internal holes of the movement object must be filled, this task is done in a Binary hole filling block, and the results are depicted in Figure 1i. Finally, a detection line must be set up in each lane on the road. For each lane, an initial terminal point is stabilized, the objects considered as movement pass through the detection line in the corresponding lane, then a buffer is initiated to store the number of foreground pixels detected. When the object is no more in the detection line, the buffer used to detect considers the absence of the object as a falling edge, and the Average value block performs a computation of the foreground pixels detected—considering the area of detection line and also the number of frames when the object was detected, then a Detection block based on threshold allows the detection and a classification of the object based in the inferred size by the values in detection line.

### 3.1. Motion Detection Using Incremental PCA

**Figure 2:**
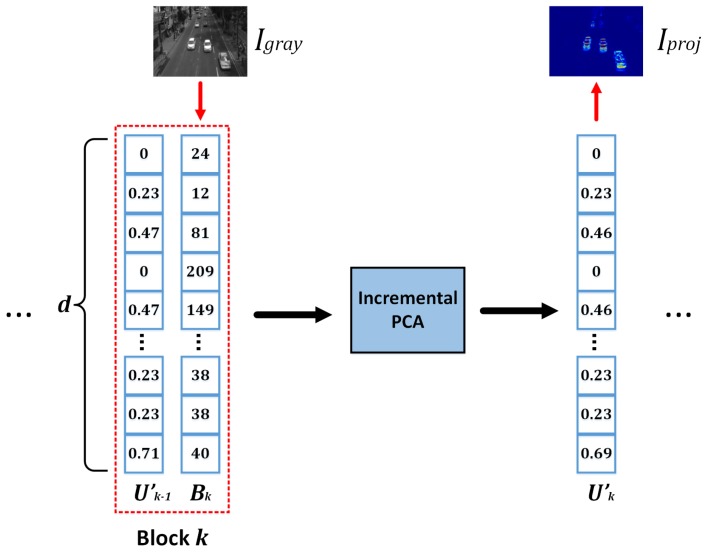
Incremental Principal Component Analysis (IPCA) process.

**Figure 3:**
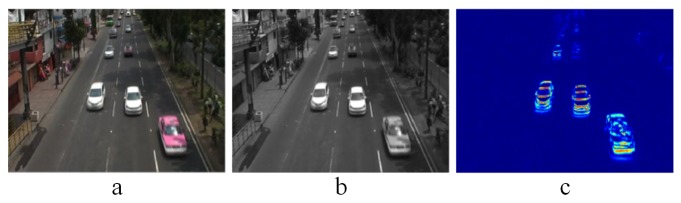
Motion detection using Incremental PCA. (**a**) Original frame. (**b**) Frame in grayscale. (**c**) Reconstructed motion frame.

As stated in Section 2, we implemented the Incremental PCA algorithm to find the first eigenvector that contains the maximum data variability between two blocks of images. For our specific purpose, blocks *A* and *B* are made up from one single frame converted to a column vector of size d×1, where *d* is the product of the width and height of the frame, that is, $d=I_{\mathrm{width}}\times I_{\mathrm{height}}$. Note that the frame width and height must be constant all along the video sequence. As initial parameters, A is set to be a zero matrix of size d×1; and *B* is set to be the first input frame of the video sequence in its vector form. Subsequently, the algorithm continues iterating for each new incoming frame. Figure 2 illustrates this iterative process and Figure 3 shows an example of a reconstructed image $I_{\mathrm{proj}}$ from the absolute value of the resulting eigenvector U′. Note that the projected image is shown using a heatmap color representation.

Another important parameter is the forgetting factor *f*. As shown in step 3 of Algorithm 1, this coefficient reduces the contribution of previous observations (frames) as new observations are available incrementally, multiplying Σ by f∈[01]. Thus, determining this coefficient is a crucial step in the Incremental PCA algorithm, since it is desirable to maintain more information about recent frames rather than the earlier ones. *f* can be manually adjusted depending on the application and the speed of the objects in motion present at the scene.

**Figure 4:**
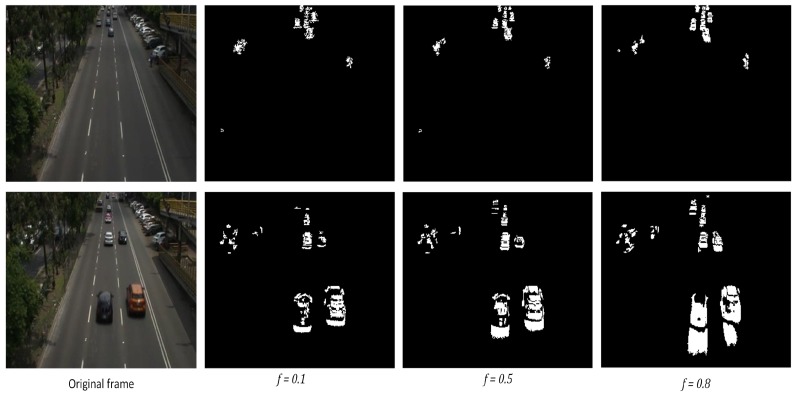
Forgetting factor effect. Upper row, *f* on small moving objects. Lower row, *f* on big moving objects.

According to our previous experiments in parallel works, a higher *f* works better for objects moving slower (e.g., pedestrians, animals, slow vehicles in streets), since the objects in motion are retained in the scene for longer intervals of time. In this application, f=0.1 has been established in order to retain just the minimum amount of movement of the vehicles that move at high speed, preventing the *“ghosting”* effect. Figure 4 shows the effect of the forgetting factor *f* for different values. It can be noticed that the higher the value of *f*, the better small moving objects in the scene are preserved. However, big moving objects leave a *“ghost”* behind them, which is not suitable for our application.

**Figure 5:**
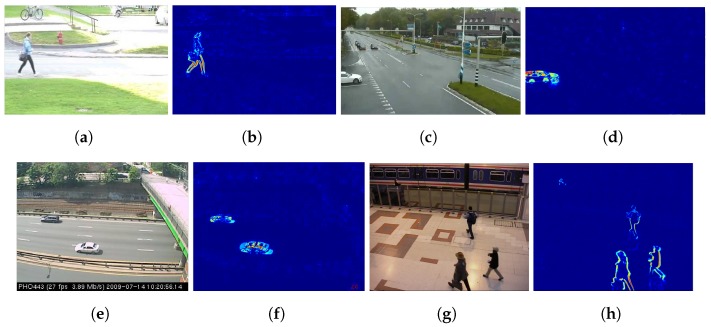
Motion detection using Incremental PCA over different scenarios from Changedetection project: (**a**) original RGB ***pedestrian*** frame; (**b**) heatmap projected of (**a**); (**c**) original RGB ***twoPositionPTZCam*** frame; (**d**) heatmap projected of (**c**); (**e**) original RGB ***streetlight*** frame; (**f**) heatmap projected of (**e**); (**g**) original RGB ***PETS2006*** frame; (**h**) heatmap projected of (**g**).

The motion detection framework, based on Incremental PCA, is able to address movement of rigid objects, such as vehicles Figure 5c–f, motor bikes, trains, etc., and also articulated movement like in pedestrian walk Figure 5a,b and Figure 5g,h. It is also able to handle different scenarios such as natural illumination or other outdoor settings, like in roads Figure 5a–f; and artificial illumination or insides, like in train stations Figure 5g–h.

### 3.2. Post-Processing

Once the projected image $I_{\mathrm{proj}}$ is obtained, a binarization process is performed using a threshold value T to obtain a new image $I_{\mathrm{bin}}$ that only contains objects in motion. $I_{\mathrm{bin}}$ is defined by:

(2) $$I_{\mathrm{bin}}\left(x,y\right)=\left\{\begin{array}{cc} 1 & I_{\mathrm{proj}}\left(x,y\right)\geq T\\ 0 & \mathrm{otherwise} \end{array}\right.,$$

where $T=2\bar{\sigma }$. We propose the use of this threshold value because, according to the literature, it contains 95% of all the information in a normal distribution of data. In this case, each frame $I_{\mathrm{proj}}$ behaves similarly to a normal distribution with mean 0 (0 value pixels indicate no motion has been detected). Approximately 5% of the remaining data in each frame is the “motion” we are interested in. $\bar{\sigma }$ is the mean of the accumulated standard deviation of all previous $I_{\mathrm{proj}}$ and is mathematically expressed as:

(3) $$\bar{\sigma }=\frac{1}{N}\sum_{i=1}^{N}\mathrm{std}\left(I_{{\mathrm{proj}}_{i}}\right),$$

where *N* is the number of frames that have been processed incrementally from the beginning, and std() is the standard deviation of the projected image $I_{\mathrm{proj}}$ at each instant *i*. std() is expressed by the following formula:

(4) $$\sigma_{X}=\sqrt{\frac{1}{N^{\prime }-1}\sum_{i=1}^{N^{\prime }}{\left(X_{i}-\bar{X}\right)}^{2}},$$

note that the input argument of std(), *X*, must be in a vectorized form. Therefore, $I_{\mathrm{proj}}$ has to be previously converted to a d×1 size vector. In addition, note that $\bar{X}$ is the mean value of vector *X* and $N^{\prime }=d$.

**Figure 6:**
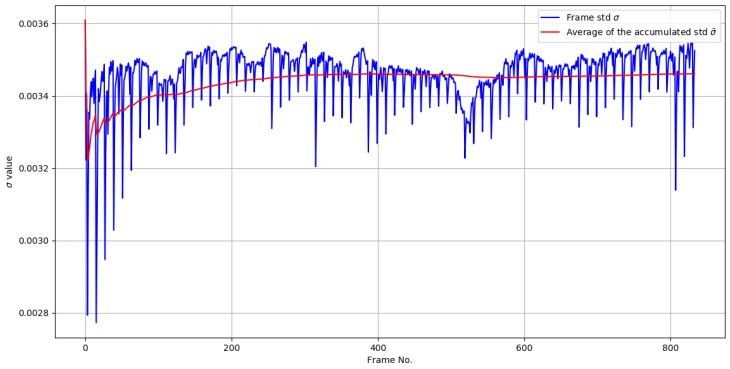
σ and $\bar{\sigma }$ values over time.

Allowing $\bar{\sigma }$ to be the dynamic component of the threshold T, we make sure that only the motion present in $I_{\mathrm{proj}}$ is preserved, removing most of the noise caused by the camera jitter, sudden illumination changes in the scene, and the noise induced by the camera itself. σ is averaged with its previous values in each new iteration for preventing abrupt changes of T, which may lead to highly noisy binarized images. This is mainly because the output eigenvector of the Incremental PCA algorithm does not contain a defined range of output values for each iteration. To illustrate this, Figure 6 shows the behavior of $\bar{\sigma }$ and σ over time for a given video sequence.

We proposed the use of $\bar{\sigma }$ due to the fact that in our testing video sequences, the vast majority of the scene is occupied by static pixels. This guarantees that most of the histogram distribution of $I_{\mathrm{proj}}$ will be centered at its mean value close to 0, indicating no motion.

After the binarization process is completed, we remove small objects in $I_{\mathrm{bin}}$ by applying a binary denoising function. This function is described in Algorithm 2 as follows:
**Algorithm 2** Binary denoising function1:Determine all the individual objects in $I_{\mathrm{bin}}$.2:Compute the area of each object in pixels.3:Remove all objects with area less than a threshold value $T_{\mathrm{bin}}$ (set their respective pixels to 0).
we have chosen $T_{\mathrm{bin}}=20$ for all our experiments.

Once the binary denoising function has been applied, we perform the OR logical operation between the current binarized frame $I_{{\mathrm{bin}}_{k}}$ and the previous one $I_{{\mathrm{bin}}_{k-1}}$. The purpose of this operation is to obtain a more “complete” version of the objects in motion present at the current frame. This logical operation is expressed by:

(5) $$I_{\mathrm{OR}}\left(x,y\right)=I_{{\mathrm{bin}}_{k}}\vee I_{{\mathrm{bin}}_{k-1}},$$

where $I_{\mathrm{OR}}\left(x,y\right)$ is the resulting binary image. This image improves the outcome of the following processing step, as we will show next.

Finally, we perform a dilatation process to $I_{\mathrm{OR}}$ using a small 2×2 structural element, then we fill all holes present to obtain $I_{\mathrm{fill}}$. A hole is basically a “dark” region surrounded by “bright” regions. In a binary image, this is translated as—dark regions (0’s) that cannot be reached through any of the edges unless we cross some bright region (1’s). This can be achieved using Algorithm 3. Consider that 0 pixel values are considered to be the background of the image.

**Figure 7:**
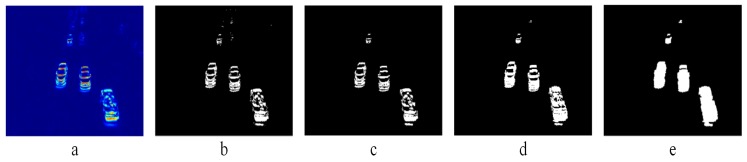
Post-processing steps. (**a**) $I_{\mathrm{proj}}$. (**b**) $I_{\mathrm{bin}}$. (**c**) $I_{\mathrm{bin}}$ denoised. (**d**) $I_{\mathrm{OR}}$. (**e**) $I_{\mathrm{fill}}$.

Figure 7 summarizes the flow of all post-processing steps, showing their individual outcomes.

**Algorithm 3** Binary hole filling
1:Apply Flood-Fill algorithm using the background edge pixels as its seed.2:Repeat step 1 until no edge background pixels exist.3:Create a mask containing only the flood-filled pixels.4:Set every non-masked pixel in $I_{\mathrm{OR}}$ to 1 to obtain $I_{\mathrm{fill}}$.


### 3.3. Vehicle Counting

**Figure 8:**
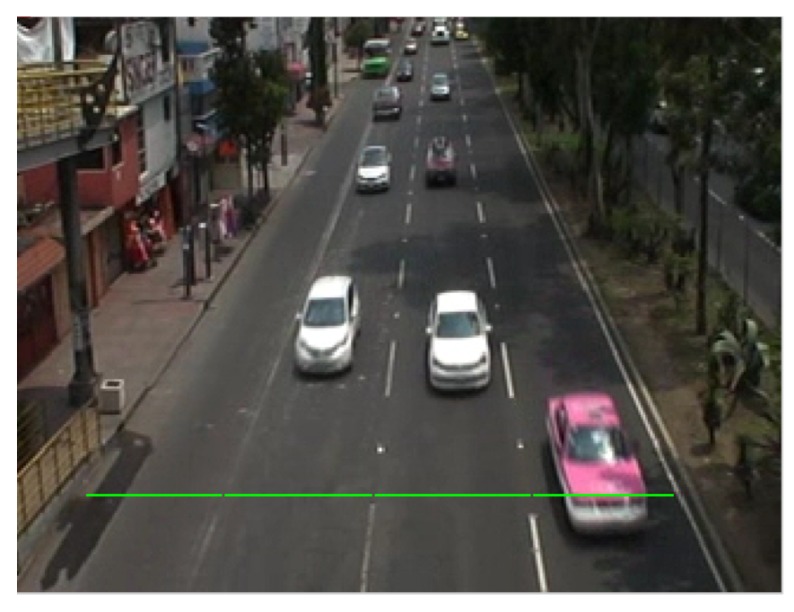
Virtual detection lines for each lane.

One of the main approaches for vehicle counting is based on extracting information using ROIs (Regions of Interests). In this work, we propose the use of a lineal ROI over each individual lane of the avenue to count vehicles. The virtual detection line is highlighted in green and shown in Figure 8.

**Figure 9:**
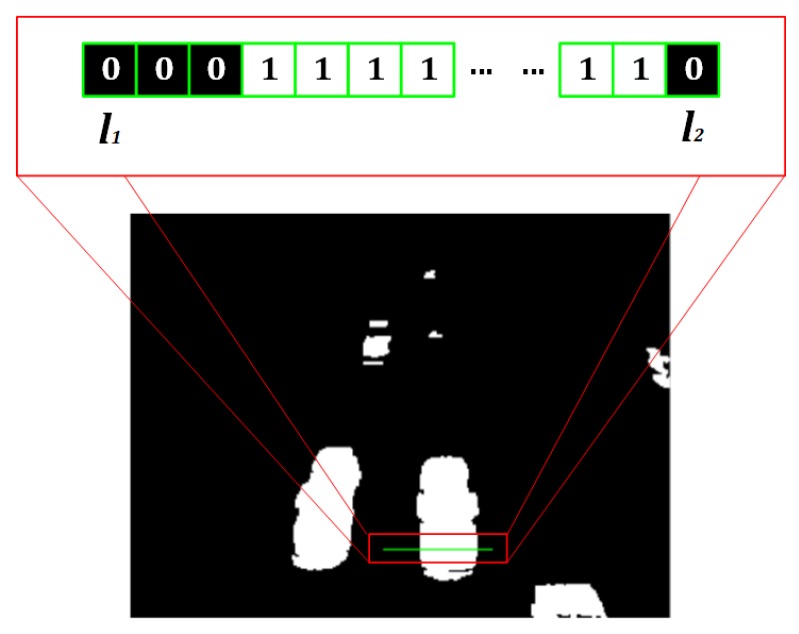
Virtual detection line representation.

Figure 9 shows the logical representation of the detection line. Pixels with value 1 are the region of the vehicle that passes through the line in the present frame; and pixels with value 0 correspond to the background.

In order to reduce the number of false positives due to large vehicles detected as multiple vehicles, we consider their presence passing through the detection line by establishing the following model:

(6) $$\mathrm{Detection}=\left\{\begin{array}{cc} 1 & \frac{1}{B+1}\sum_{i=k-B}^{k}\sum_{y=l_{1}}^{l_{2}}I_{{\mathrm{bin}}_{i}}\left(x,y\right)\geq T_{\mathrm{count}}\\ 0 & \mathrm{otherwise} \end{array}\right.,$$

where, Detection is a logic variable that indicates if a vehicle exists or not in the detection line, $I_{\mathrm{bin}}\left(x,y\right)$ is the image containing the vehicles in motion, $l_{1}$ and $l_{2}$ are the initial and final columns of the detection line for a fixed row *x* of $I_{\mathrm{bin}}\left(x,y\right)$, *B* is the number of consecutive frames used to compute the mean value of the area occupied by the vehicles, and $T_{\mathrm{count}}$ is the threshold value used to discriminate between noise and actual vehicles [15].

**Figure 10:**
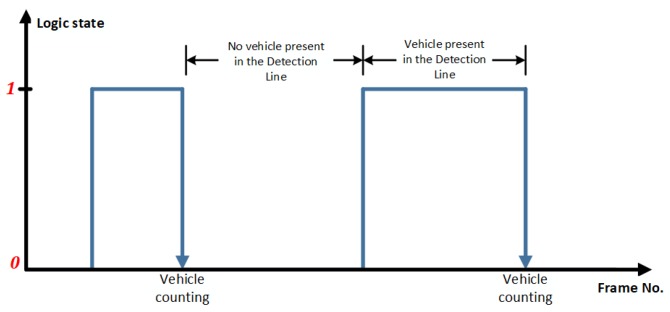
Vehicle counting by detecting falling edges.

Each individual vehicle is counted only when a falling-edge frame is detected followed by a previous detection of a rising-edge frame. This process is illustrated in Figure 10.

## 4. Results

**Figure 11:**
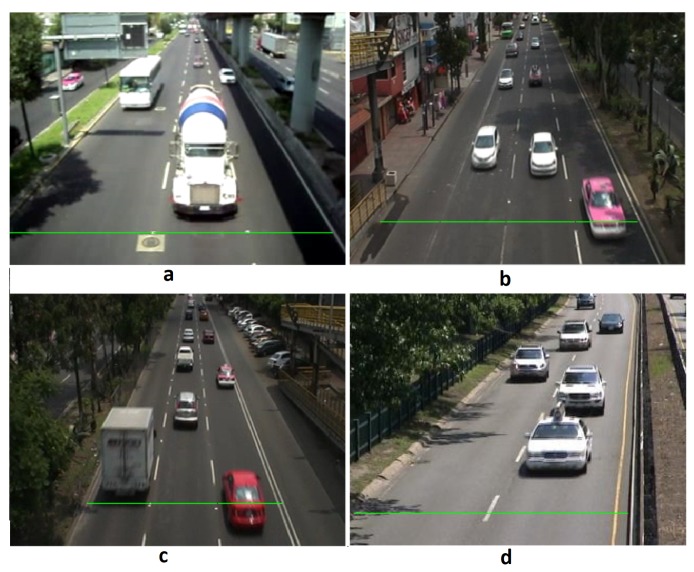
Testing video sequences. (**a**) Video No. 1, (**b**) Video No. 2. (**c**) Video No. 3. (**d**) Video No. 4.

We evaluated the effectiveness of our proposed methodology by using four video sequences. The first three sequences were recorded by ourselves during the daytime in different places in Mexico City. The first two sequences show vehicles transiting towards the camera position and the third one shows vehicles transiting in the opposite direction. The last video sequence, called *Highway* was taken from Changedetection project, which is a website that summarizes an academic benchmark for testing and ranking existing and new algorithms for change and motion detection, providing several datasets and tools [36]. Previews of the four video sequences are shown in Figure 11.

We recorded our three video sequences using a Sony DCR-SR100 video camera. Video No. 1 was recorded using automatic light exposure. This video presents camera jitter, especially when large vehicles transit below the pedestrian bridge where the camera was placed. This video also contains a concrete mixer truck and a large public service vehicle. Due to the auto exposure configuration, artificial illumination changes are induced when those large vehicles are present, this issue arises not only when large vehicles are in the scene, but also alongside the foreground aperture problem. The camera jitter and artificial illumination changes are settled by the IPCA motion detection framework, and the foreground aperture problem is address by post-processing stages described in Section 3.2, particularly in the frame fusion block in Figure 1. Videos No. 2 and No. 3 were recorded using the same video camera, but configured with manual light exposure, so that no changes in illumination are induced by big brilliant vehicles. In Video No. 2, the traffic flow is from the bottom frame to the top frame, the set of this experiment is to evaluate the effectiveness of the vehicle counting process when the object is decreasing its relative size, the results demonstrate that our framework can address this scenario. Video No. 2 and Video No. 3 presents a small amount of camera jitter and a gradual environmental illumination change caused by a cloud passing by, in both cases, the IPCA motion detection framework is able to manage these issues. In video No. 3, the detection line in the most right lane is in the shadow of some bushes, this induces changes due to natural movement in the leaves and branches, but the combination of the implemented motion detection based on IPCA and the detection line allow the proposed method to tackle the problems related to inference in motion detection caused by the shadows of the bushes. In Video No. 4, the angle between the traffic flow and the camera plane is slightly different than the other videos, this configuration can overlap the movement objects in independent lanes, especially if the angles increase. In order to avoid this problem in counting vehicles, the camera position should be set perpendicular between the camera plane and the traffic flow. All four videos were previously converted to individual frames of size 320×240 for convenience of analyzing individual frames of the sequence. This size normalization implies $I_{\mathrm{width}}$ = 320 pixels, $I_{\mathrm{height}}$ = 240 pixels, and finally $d=I_{\mathrm{width}}\times I_{\mathrm{height}}$ = 76,800 pixels.

**Figure 12:**
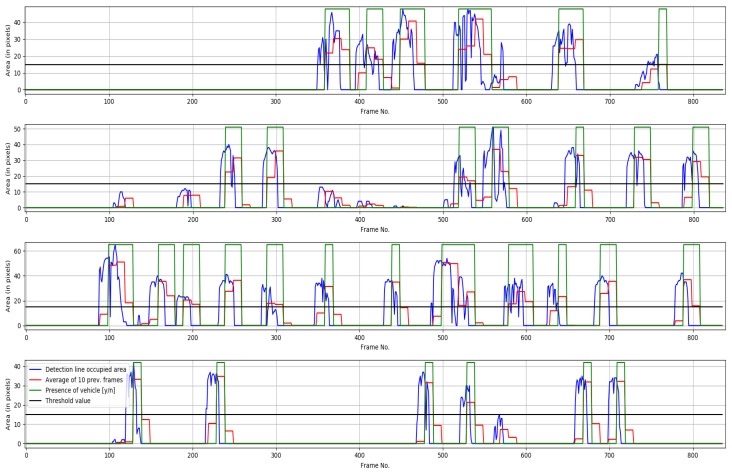
Counting results for video sequence No. 2. From top to bottom: Lane #1, Lane #2, Lane #3, Lane #4.

As for the actual counting component, Videos No. 1 and No. 3 count vehicles in three lanes of the road, Video No. 2 does it in four lanes and Video No. 4 does it only in two lanes. Figure 12 shows an example of the behavior of the counting process as the frames are processed sequentially in lane #3 of video No. 3 with B=10.

Table 1, Table 2, Table 3 and Table 4 show the final performance of our system for each video sequence, presenting their corresponding rate of detection, false positives, false negatives, and accuracy for each road lane (from left to right).

On average, the entire process runs at 26 frames per second (fps) on a standard 2.0 GHz dual core PC. Similarly, from Table 1, Table 2, Table 3 and Table 4 it can be shown that the system average accuracy was 96.6%.

## 5. Discussion

Intelligent transportation systems are currently becoming very important and will definitely play a vital role in smart cities of tomorrow. Specifically, vehicle counting is of great importance for many real world applications, such as urban traffic management. Several methodologies have been proposed in order to improve the overall quality, performance, efficiency, and cost of this kind of systems. Our proposed methodology only addresses the problem of counting vehicles under some of the most common problems, such as small camera jitter and illumination changes due to the environment or the camera auto exposure time. We acknowledge that there exists an immense number of problems and challenges yet to be solved. However, related works have also addressed very specific challenges since no general solution exists. In Table 5, we try to summarize as briefly as possible related works by their performance in terms of accuracy, fps, and type of hardware used. Similarly, in Table 6 we show some comments about the related works.

## 6. Conclusions

In this paper, we presented a methodology based on incremental subspace learning for detecting changes in consecutive frames of video sequences. The resulting vector of this incremental learning process is reconstructed into an image. This image is then post-processed for detecting regions where motion is present. Finally, a statistical algorithm based on the average value of the frames is used to determine the presence of vehicles and also to count them. Our proposed methodology has proven to be useful in real scenarios (as described in the Results section) where light conditions change over time due to the environment and also due to the camera auto exposure. Moreover, it can also handle small camera jitter during several continuous frames with no additional filtering. It is clear that our specific application of Incremental PCA is somehow similar to the frame differentiation methodology for motion detection. However, we make a clear distinction performing a *statistical* difference between a frame made up from previous accumulated observations and the current one. Additionally, the fact that the forgetting factor *f* can “discriminate” earlier observations (frames) to a lesser or greater extent makes this methodology flexible for different applications as it provides an improved version of a standard frame differentiation methodology. Experimental results have demonstrated that, in most cases, our methodology is able to count vehicles effectively with up to 100% accuracy, while preserving an optimal performance in fps, suitable for real-time implementation. In future works, descriptive algorithms can be implemented for detecting vehicles given proposed regions of objects in motion in order to perform a more robust and complete segmentation. Lastly, our future scope is to apply Deep Learning models for performing vehicle classification and to mine data for security and video surveillance purposes.

## Figures and Tables

**Table 1:** Counting results for Video No. 1.

Video No. 1	Detected Vehicles/ Total Vehicles	False Positives	False Negatives	Accuracy
Lane #1	9/9	0	0	100%
Lane #2	10/10	0	0	100%
Lane #3	13/13	0	0	100%
**Total**	32/32	0	0	100%

**Table 2:** Counting results for Video No. 2.

Video No. 2	Detected Vehicles/ Total Vehicles	False Positives	False Negatives	Accuracy
Lane #1	6/6	0	0	100%
Lane #2	7/7	0	0	100%
Lane #3	12/12	0	0	100%
Lane #4	6/7	0	1	85.71%
**Total**	31/32	0	1	96.87%

**Table 3:** Counting results for Video No. 3.

Video No. 3	Detected Vehicles/ Total Vehicles	False Positives	False Negatives	Accuracy
Lane #1	7/7	0	0	100%
Lane #2	15/13	2	0	84.61%
Lane #3	10/10	0	0	100%
**Total**	32/30	2	0	93.33%

**Table 4:** Counting results for Video No. 4.

Video No. 4	Detected Vehicles/ Total Vehicles	False Positives	False Negatives	Accuracy
Lane #1	17/17	0	0	100%
Lane #2	9/10	0	1	90%
**Total**	26/27	0	1	96.29%

**Table 5:** Comparative analysis between related works.

Method	Accuracy	fps	Hardware
Liu, F., et al. [25]	99%	10 fps	Not reported
L. Rosas-Arias, et al. [15]	100%	Not reported	2.0 GHz Intel CPU
Mundhenk T.N., et al. [21]	Not reported	1 fps	Nvidia Titan X GPU
N. Seenouvong, et al. [16]	96%	30 fps	2.4 GHz Intel CPU
N. Miller, et al. [13]	93%	Not reported	Not reported
J. Quesada, et al. [12]	91%	26 fps	3.5 GHz Intel CPU
J. Zheng, et al. [14]	90%	Not reported	3.2 GHz Intel CPU
Ahmad Arinaldi, et al. [22]	70% (at most)	Not reported	Not reported
**Ours**	96.6%	26 fps	2.0 GHz Intel CPU

**Table 6:** Comments about related works.

Method	Comments
Liu, F., et al. [25]	Reaches 99% of accuracy only under ideal situations.
L. Rosas-Arias, et al. [15]	Reaches 100% of accuracy only under ideal situations.
Mundhenk T.N., et al. [21]	High aerial coverage area. Vehicles are counted as individual hi-res images.
N. Seenouvong, et al. [16]	Does not update the background model and is not robust to illumination changes.
N. Miller, et al. [13]	The counting process uses a very complex configuration of ROIs.
J. Quesada, et al. [12]	Utilizes an incremental approach for detecting motion in aerial images (top-view).
J. Zheng, et al. [14]	Although it is not reported, authors claim their proposed method runs in real-time.
Ahmad Arinaldi, et al. [22]	The system is evaluated under both standard and very challenging environments.
**Ours**	Balanced methodology between accuracy, fps, hardware, and robustness.
